# Dapagliflozin-Based Therapies in Patients With Type 2 Diabetes Mellitus in an Outpatient Real-World Setting Across India: The DAPATURN Study

**DOI:** 10.7759/cureus.106506

**Published:** 2026-04-06

**Authors:** Rajeev Chawla, Sabeer T. K., K. P. Singh, Ashwani Mehta, Mounam Chattopadhyay, Kaushik M Rakshit, Nikhil Motiramani, Rajeev Philip, Amber C Shinde, Sowmiya M K., Santosh Revenkar, Kamlesh Patel

**Affiliations:** 1 Diabetology, North Delhi Diabetes Centre, New Delhi, IND; 2 Diabetes and Endocrinology, Diacare Centre for Diabetes and Endocrinology, Kannur, IND; 3 Diabetes and Endocrinology, Fortis Hospital, Mohali, IND; 4 Cardiology, Sir Ganga Ram Hospital, New Delhi, IND; 5 Diabetes and Endocrinology, Techno India Dama Hospital, Kolkata, IND; 6 Cardiology, Shaw’s Diagnostic, Kolkata, IND; 7 Medicine, Shri Balaji Institute of Medical Sciences, Raipur, IND; 8 Endocrinology, Pushpagiri Institute of Medical Science and Research Centre, Thiruvalla, IND; 9 General Medicine, Health First Clinic, Mumbai, IND; 10 Medical Services, Medical Affairs Division, Lupin Limited, Mumbai, IND

**Keywords:** comorbidities, dapagliflozin, india, sitagliptin, usage pattern

## Abstract

Aim: Determination of usage patterns and clinical profile of patients receiving dapagliflozin and its combinations in outpatient settings across India.

Methods: An observational, cross-sectional study was conducted across 661 sites in four zones of India from March to December 2024. Baseline demographics, comorbidities, and used dapagliflozin formulations (dual or triple fixed-dose combination (FDC)) were recorded to analyze usage patterns.

Result: A total of 34,443 patients were enrolled from North (*n* = 8,035), South (*n* = 8,205), East (*n* = 11,143), and West (*n* = 7,060) India, with a mean age of 54.94 ± 11.35 years. Mean glycemic hemoglobin (HbA1c) and body mass index were 8.53 ± 1.56% and 27.21 ± 4.93 kg/m², respectively. The most common formulation was dapagliflozin + sitagliptin + metformin triple FDC (*n* = 11,657, 33.84%), followed by dapagliflozin + sitagliptin dual FDC (*n* = 9,940, 28.86%) and dapagliflozin + metformin dual FDC (*n* = 1,808, 5.24%). Dapagliflozin + sitagliptin was most used in patients with hypertension (*n* = 1045, 34.30%), dyslipidemia (*n* = 500, 35.06%), chronic kidney disorder (CKD) (*n* = 54, 36.99%), heart failure (HF) (*n* = 52, 35.86%), hypertension + dyslipidemia (*n* = 225, 31.91%), and atherosclerotic cardiovascular disease (ASCVD) + dyslipidemia (*n* = 124, 38.15%). Triple FDC use was highest in ASCVD (*n* = 782, 33.56%) and HF + CKD (*n* = 15, 50%). In both HbA1c groups (<8.5% vs. >8.5%), dapagliflozin + sitagliptin (*n* = 6,332, 31.5%, vs. *n *= 3,608, 25.16%) and triple FDC dapagliflozin + sitagliptin+ metformin (*n* = 6,456, 32.11%, vs. *n* = 5,200, 36.27%) were most used. Dapagliflozin + sitagliptin use increased with diabetes duration (<1 year: *n *= 420, 23.4%, to >10 years, *n *= 1,422, 39.45%), while triple FDC preference remained high from <1 year (*n* = 637, 35.49%) to 10 years (*n* = 4,192, 34.96%).

Conclusions: This study highlights real-world dapagliflozin use in India, showing that dapagliflozin-based FDCs are widely adopted across diverse patient groups.

## Introduction

Diabetes is a chronic metabolic disorder characterized by persistent hyperglycemia resulting from impaired insulin secretion, insulin action, or a combination of both [1]. Globally, an estimated 589 million adults aged 20-79 years are living with diabetes, making it a major public health challenge [2]. Type 2 diabetes mellitus (T2DM) accounts for nearly 90% of all diabetes cases and contributes substantially to morbidity, mortality, and healthcare costs [2,3]. Several risk factors predispose individuals to T2DM, including ethnicity, family history, advancing age, overweight and obesity, unhealthy diet habits, physical inactivity, smoking, excess body fat, and higher body mass index (BMI) [4]. The disease is associated with both microvascular complications (retinopathy, neuropathy, and nephropathy) and macrovascular complications (cardiovascular disease (CVD), chronic kidney disease (CKD), cerebrovascular, and peripheral vascular disease) [5]. Earlier studies have shown that prolonged hyperglycemia significantly increases the risk of these complications, underscoring the importance of early and effective disease management [6,7].

Current management strategies for T2DM focus on lifestyle modification, dietary interventions, and pharmacological therapy [8]. Among pharmacological agents, sodium glucose cotransport 2 (SGLT2) inhibitors have shown benefits in glycemic control and in reducing cardiovascular and renal risk. By lowering glucose reabsorption in the kidneys, they improve glycemic control while also promoting weight loss, blood pressure reduction, and osmotic diuresis, thereby contributing to favorable cardiovascular outcomes [9].

Dapagliflozin, an SGLT2 inhibitor that has been approved for the treatment of T2DM [10]. When used in combination therapy, dapagliflozin has shown effectiveness in improving glycemic control in patients with uncontrolled diabetes, while also reducing the risk of cardiovascular death, hospitalization for heart failure (HF), and progression of renal disease [10]. With the rising prevalence of T2DM in India, it is essential to generate real-world evidence on treatment practices. Accordingly, the present study was conducted to evaluate the usage patterns and clinical profiles of patients receiving dapagliflozin and dapagliflozin fixed-dose combination (FDC) therapy in outpatient settings across India.

## Materials and methods

Study design

The observational, cross-sectional study was conducted across 661 sites in four zones of India from March 10 to December 20, 2024 (Table 1).

**Table 1:** List of participating sites. Each listed healthcare professional (HCP) represents an individual participating study site.

Sr. no.	HCP name	City
1	Rose Mary G., Shameer Basheer Kunju, Rajeev Philip, Sabeer T. K., Sreejith M. G., Jiljith K., Iyad Mohamed P., Sabeer A. Rasheed, Manjunatha Kurugodumath, Nagesh Manohar Prabhu, Binish Sreekumar, Deepak Raju, Abdhul Kareem A. K., Bibin Jacob, P. Praveen Kumar, Rajagopal Rajeev, Rakesh Kumar Jha, Sreejith M. G., Rajalakshmi S., Sreeraj S.	Trichur
2	Sudip Chowdhury, Rajarshi Banerjee, Suman Saha, Victor Saha, Mounam Chattopadhyay, Kaushik Mohan Rakshit, Chanchal Kumar Dalai, Ayan Mukherjee, Indranath Banerjee, Apurba Basu, Deblina Sarkar, Joydeep Bhowmick, Chandan Kumar Saha, Rudra Paul, Tanmay Diasi, Sunil Kumar Lhila, Santu Biswas, Ankur Dasgupta, Utsa Basu, Mohammad Tahir Ansari, Arkaprava Dasgupta, Satyabrata Mukherjee, Sourav Mukerji, Tanmoy Majee, Trithankar Sarkar, Arcojit Ghosh, Souvonik Mandal, Sabyasachi Roy, Arunava Ghosh, Sudip Kumar Pore, Arjun Kumar Paul, Debarati Bhar, Ritam Roy, Anwar Jamal, Tapas Bakuli, Paramita Bhattacharya, Niharendu Das, Sabyasachi Roy, Rishad Ahmed, Abhijit Roy, Sanjib Kumar Patra, Khan Obydul, Shubhagata Chaudhury, Radheshyam Agarwal, Md Gousul Alam, Debojyoti Bhattacharjee, Dibyendu Dalui, Sandeep Lahiry, Sudipta Pramanick, Shahid Haider Warsi, Swatilekha Das, Sayan Mazumder, Soumyajit Ghosh, Deep Goswami, Manoj Kumar Yadav, Ranadhi Das, Joydip Datta, Koushik Majumdar, Ranadeb Roy, Sourav Bhattacharjee	Kolkata
3	Srijit Chatterjee, Abu Md Mustaque, Banibrata Bera, Arijit Kundu, Subrata Chakrabarti, Tamoghna Maiti, Rakesh Kumar, Ritendra Nath Talapatra, Shouvik Choudhury, Sandeep Kumar Ratha, Santosh Kumar Dey, Supriya Ghosh, Sandip Kumar Das, Md Shah Alam, Sanjib Kumar Pal, Suman Kumar Ghosh, Kalimujjaman Molla, Subham Das, Subham Das Goswami, Vineet Kumar, Mahesh Rath, Apurba Ranjan Jena, Subhro Pandey, Abhirup Basak, Shibaditya Chakraborty, Subrata Bhattacherjee, Rik Swarnakar, Ardhendu Sahana, Sougata Ghosh, Sandipan Mistry, Saikat Das, Vikram Agarwal, Surojit Banerjee, Sekh Sarafat Hossain, Tarak Nath Chattopadhyay, Shankha Ghosh, Diptiman Sahoo, Sanjib Kumar Lenka, Pradeep Kumar Panigrahi, Sayed Ikbal Hussain, Abhisek Tripathy, Ashish Kumar Jain, Ranajit Kumar Panda, Lakshmi Kanta Dalbera, Rajesh Maji, Chandan Das, Raman Raj, Amitabh Biswas, Shubhrangsu Samanta, Shubhankar Mukherjee, Sudipta Joy, Sibaprakash Mukherjee, Dibyajyoti Gupta, Santhini P Bishal, Chaube Anand Akhilesh, Satyaki Roy, Goutam Banerjee, Swarnasindhu Bhowmik, Sandip Kumar Das, Samya Dutta, Kundan Singh, Abu Md Mustaque	Burdwan
4	Manish Bothale, Ramesh Malkar, Prakash Bikkad, Shekhar Ramchandra Bhise, Vaishali Kasare, Rohan Kate, Suruchi Mandrekar, Ankur Sharma, Sagar Idhol, Ravindra Ramhari Sangolkar, Sachin Rajaram Gavade, Rajeshwar Supekar, Anuj Darak, Pavan Agrawal, Amar Amale, Amit Kothari, Aakash Badgujar, Sagar Shrirao, Ninad Bhosale, Rajeshwar Supekar, Akshay Dalal, Amit Palve, Pratik Barai, Kiran Madhukar Somkumar, Aziz Khan, Amit Palange, Kevin Bora, Nitin Wadaskar, Satish Ramkisan Sawant, Vaibhav Deshmukh, Rahul Kadu, Sushant Gune, Yogeshvilasrao Salunkhe, Akash Badgujar, Shrikant Kale, Nitin Sonune, Alok S. Shinde, Annapoorna Ajit Kalia, Chadha Rohitsingh Mohinderpalsingh, Prakash Shende, Rohit R. Shriwastav, Sandeep M. Sonone, Shrikant Lakshmikant Bhoskar, Swanand Lunge, Wagh Vrushali Rajesh, Sattyjit Borade, Hemant Mahesh Manjrekar, Chetan Rathi, Abhay Mali, Manish Ambadkar, Zubair Zafar Khan, Umesh Badhe, Sudhanwa Deepak Kulkarni, Aniket Ajit Oswal, Gajendra Agrawal, Prafulla Sunil Kabra, Deepankar Mishra, Ritesh Vishwakarma, Wagh Vrushali Rajesh, Prasad Shrinivas Korulkar, Harshavardhan Narute, Suyog Chopade, Swapnil Lahole, Reshma Kshirsagar, Rohit Jakhotia, Swaroop Verma, Ayachit Kesharwani, Deepak R. Nenwani, Ratnakant Magdum, Surekha Mahesh Patil, Akshay Kashid, Suruchi Mandrekar, Deepankar Mishra	Pune
5	Kiran Pal Singh, Abhishek Mittal, Rakesh Luthra, Jagdish Kumar Bhutani, Resham Singh, Anshuman Gupta, Ashdeep Singh, Varinder Mahi, Bhupender Kumar, Anuj Kamboj, Suraj Shoor, Anu, Venkateshan, Nitin Mittal, Raman Arora, Hemant Puri, Kamaldeep Bansal, Arvind Sharma, Ashuootosh Bhardwaj, Rajesh Kumar Sharma, Swati Soni, Anil Kumar, Sonu Kumar Singla, Jahangir Rashid Beig, Manjeet Singh Trehan, Harbir Kaur, Birinder Singh Gandhi, Varun Kumar Mahajan, Irfan Gul Beigh, Geetika, Vinay Vedvyas, Suraj Agrawal, Sandeep Suri, Nitesh Kumar, Vishal Sharma, Sajan Kumar Sain, Ashok Sama, Kusum, Ankur Gupta, Mohd Vaseem Akram, S. Gunasekaran Sambandam, Hitender Kumar, Vikram Kumar, Aftab Ahmed Aftab, Saurabh Sharma, Syed Afaq Jalali, Vinod Kumar, Ankit Chhabra, Sidharth Arora, Sunil Bansal, Balraj Gupta, Damanjit Singh, Guruvinder Singh, Yash Paul Mehra, Atul Mehta, Rashmeet Singh, Revanasiddappa, Shivam Sharma	Chandigarh
6	Amol Manerkar, Amber Shinde, Ankur Patel, Ambari Firdoush Shaikh, Vivek Agarwal, Poonam Tuteja, Shreyas Deepak Wajekar, Anisha Shah, Rahul Nikumbhe, Kanhaiyalal Kumbhakar, Gaurav Surana, Prasoon Srivastava, Newton Ghosh, Tanay Ajit Padgaonkar, Aashna Patil, Sonu Kumar Puri, Azaz Khan, Jayesh Nirmalkumar Thaddani, Siddharth Pankaj Kumar Singh, Makarand Sudhakar Salunke, Bhondve Amit Sudarshan, Shradha Rushabh Doshi, Pinkesh Shashikant Rajput, Ameya Patil, Hardik Bambhania, Ankeet Dedhia, Madhu Gupta, Shoeb Nizamuddin Kardekar, A Dasgupta, Kshitij Balwalk, Sailee Patil, Vipin Pannalal Dubey, Aniket Chandrakant Mandrekar, Alpesh Jain, Jagdale Ganesh Subhash, Aditya Dhananjay Phadte, Aparna Gangurde, Vishakha Mhatre, Sanjay Rajdev, Kirankumar Bhikkad, Indumathi Thanga Kuberan, Soumya Poduval	Mumbai
7	A. Rufus Rajadurai, R. Anitha, Manivarmane R., J. P. Vignesh Ramanan A., K. Sivaprakash, M. Natarajan, Ranjith Pratap S., Md Nayeemuddin, B. Anandakumar, Premnath, A. Ganesh Raja, M. Natarajan, Bala Vignesh Raman, Mubarak Raja, T. V. Rajesh, R. Sasinthar, D. Parameswaran, R. Gopalakrishnan, A. Mohammed Meeran, X. Rufus Demel, M. Dhanasekar, R. Simsonraj, J. Innisai, K. Saminathan, Jonathan Vivek M., Lawrence Victor Joe, Ashok Kumar T., S. Antony Prince Amalan, Dilip Sathya Kumar, V. Pranesh, J. Vinoth Kumar Philip, K. Prasad Kumara, Siva Ranganathan Green, Siddharth P., B. Ramesh Babu, A. Saravana Pradeep Kumar, Tamil Azhagan B., M. Burose Khan, Mohamed Faizal S. M., A. Moorthi, K. Kanagasanthosh, Ram Vivek K., N. Karthik, S. Suresh Kanna, Subramanian K., M. Venkatesh, R. Srinivasan, M. Ashok	Chennai
8	Arun Kumar Paharia, Rajat Kanti Bhattacharjee, Bhaskar Brahma, Umar Faruque, Brijes Saha, Sumit Sarkar, Gaurav Kumar Singh, Ranjan Bhowmik, Hemant Kumar Garg, Rana Majumdar, Mizan Ahmed, Arup Mondal, Upakul Bora, Mrinal Joyti Bora, Kamalika Das, Rajib Borkataky, Partha Roy, Kamal Kumar Jain, Asaduz Zaman Ahmed B., Mridul Mahanta, Mridusmita Khataniar, Debasish Paul, Darpan Gogoi, Indra Kuladhipati, Ramthaipou Kamei, Subhamay Ghatak, Jhutan Chowdhury, Sandip Sarkar, Arkapratim Chowdhury, Syed Shayeb Mahmud, Rinku Dutta, Pranjal Deori, Biswadeep Dhar, Navin Kumar Bansal, Anjan Jyoti Talukdar, Panchanan Uzir, Bhaskar Thakuria, Bikash Rai Das, Meraj Uddin Ahmed, Pankaj Chaudhary, Pramath Das, Rupam Choudhury, Bhaskar Neog, Pranpratim Saikia, Deepjyoti Saikia, Ankit Sharma, Nadim Ahmed, Mohammed Eijaz, Ahmed Laskar, Sri Pranab Kumar Bordoloi, Sudipto Saha, Feroz Hussain, Prashanta Dey, Huidrom Manimohon Singh, Nitesh Pansari, Saumen Chaudhuri, Sanjoy Kumar Saha, Pranab Kanti Datta, Dipul Rudra Paul, Rajkumar Bhattacharjee, Naveen Kumar Agarwal, Srikumar Biswas, Arijit Saha, Mrinmoy Adhikary, Dipayan Paul, Puspendu Sardar	Guwahati
9	Ashish Patel, Shweta A. Bhanot, Sudeep Jain, Nikhil Motiramani, Devendra Kumar Sahu, A. A. Ansari, Parag Nilkanth Manape, Abhimanyu Nigam, Shireesh Mishra, Satish Choudhary, T. R. Rathore, Animesh Choudhary, Rupesh Shrivastava, Deepika Bhagat, Prakash Jaiswal, Jinesh Kumar Jain, Pritesh Masih, Vishal Gourh, Rajesh Kumar, Jambu Kumar Jain, Satish Choudhary, Ashish Patel	Indore
10	Vallabhaneni Krishna Chaithanya, Rakesh Tumula, Pilla Rakesh, Maddukuri Hanumantharao, Rajeev Boreddy, L. K. V. Kumar, Yugandhar Mamidi, Mohammad Saleem, Jampani Pardhasaradhi, Krishna Prithvi Boggavarapu, Baby Nagapriya Vellalacheruvu, Kiran Kumar Golla, Vivek Athunoori, D. S. C. Bhaskar, Veera Balaji Joga, V. K. Chaitanya, Srikanth Bodepudi, Sumalatha, Loknath Tripathy, Kakani Venkata Surendra, Tirunamalli Venkateswarlu, Mohd Riyaz, Mohammed Rasheed Ali, Samir Kumar Panda, Araveti Jeswanth, S. Nageswar Rao, Chillara Durganadh, Srikanth R., Nuthakki Vijaya Lakshmi, Mohammed Yousuf Khan, Sowjanya Reddy Bhoopthi, Lavanya Vodlakonda, Paripally Anish Reddy, Tudi Pavan, Kiran Satla, Shehazad Ruman	Hyderabad
11	Dheeraj Kumar Singh, Manish Shrivastav, Upali Nanda, Ashish Jain, Sidhant Singh Thakur, Gaurav Mittal, Vineet Jain, Mayank Uppal, Ashwani Mehta, Abha Mishra, Ritesh Jain, Nehal Nischal Vora, Sasanka Kumar Basu, Kapil Khanna, Anil Laul, Amit Prakash Singh, Ajit Kumar Talukdar, Mohammadd Ashraf Khan, Saurabh Sharma, Dharmander Singh, Pooja Garg, Vageesh Kathuria, Deepak, Deepak Devgun, Subodh Kansal, Himanshu Bhatheja, Manik Gedam, Gautam Sharma, Rajeev Chawla, Sanjay Kumar Gogia, Manish Gupta, Jai Khullar, Manish Kumar Sharma, Harish Takkar, Harjit Singh, Yogendra Singh Rajput, Atul Saxena, Deepak Kumar, Ashish Jain	Delhi
12	S. Sandeep, N. S. Ramesh, Shrimanth Y. S., Karibasaiah A. M. A., Sreedhara R., Jayasheelan M. R., Kumar P., Prashanth G., Dinesh Joshi, Jagadish Chandrashekhar Shirol, Samarth S., Nikhil Karekannapal, Vijaykumar J. R., Saiyadali A. Allisabanavar, Pooja Shankar S., B. S. Nagaprakash, Basavaraj S. Kumbar, Sandeep Ijanthkar, Nitesh D. Jain, Praveen Kumar Kusubi, Nidhin Mohan, Hosakere Nagaraj Rajendrakumar, Raghu G., A. R. Renukaprasad, Yallappa Reddy H. M., Imran Damani, Pavan P. Rasalkar, Darshana, Sagar Patil, Mohammed Tauseef, Shriharish B. Pujari, Vishal Kumar H., Mujeeb Nasrulla	Bangalore
13	Abhinav Rastogi, Vishal Virendra Singh, Parul Singhal, Vikas Juneja, Yogendra Babu Agarwal, Abhishek Kumar, Rajesh Chaurasiya, Vivek Gupta, Hari Om, Geetesh Manik, Ajeet Singh Chahar, Jatin Garg, Shubham Shekhar Singh, Jitendra, Kumar Doneria, Rakesh Kumar, Gurudatta Maharana, Harish Chandra Lamba, Aashaq Hussain Khandy, Anup Kumar, Saurabh Kumar, Gaurav Gangwar, Sumit Kant Jha, Vineet Jain, Amit Goyal, Rajiv Kumar Gupta, Pankaj Kumar, Vjay Singhal	Ghaziabad
14	Sanjeev Kumar Pandey, Umesh Kumar Sah, Divakar, Akhilesh Tripathi, Ankur Poddar, Devendra Nath Sharma, K. Tripathi, Ritesh Khanna, Abhishek Arun, Mohit Mohan Singh, Nikhil Sinha, Yashpal Singh, Ankur Poddar, Prashant Kumar Singh, Prashant Dwivedi, Manish Dwivedi	Lucknow
15	Shiv Prakash Sharma, Sourav Bansal, Vineet Kumar Jain, Niharika Sharma, Swati Srivastava, Diwanshu Khatana, Manoj Kumar Jain, Himanshu Agarwal, Suvagiya Ashwin Kalubhai, Kirit Narottamdas Kubavat, Paras Mal Jain, Abdul Tanvir Khan, Niranjan Agarwal, Rajnish Saxena, Ankit Rathi, Ramkesh, Rishi Kumar Tailor, Amit Jakhar, Shah Moxit Sunil Kumar	Jaipur
16	Saket Vats, Chandan Kumar, Raj Vardhan, Balram Kumar Singh, Sudipta Kumar Rout, Manish Kumar, Bishwajit Pd Azad, Nirmal Kumar Jain, Rajnish Kumar, Danish Akhter, Kumar Durgeshwar, Kumar Alok, Tusharkanti Sahu, Sachin Kumar, Khawaja Ahtesham Ahmad, Rajeev Ranjan, Nagendra Mohan Sinha, Kewal Sharan, Devesh Chatterjee, Rakesh Kumar, Pankaj Kumar, Mohammad Anay Tulla, Ram Babu Sharma, Pradeep Kumar, Aman Sinha, Gautam Sanjay Kumar, Rajeev Ranjan, Ravi Maharshi, Manish Kumar, Mohit Narayan, Narendra Jha, Sameer Kumar Mehta, Syed Hedayetullah, Sibaram Panda, Santosh Kumar Tete, K. D. D. Bibek, Mukesh Kumar Agarwal	Patna

The study was conducted in accordance with the International Council for Harmonization Good Clinical Practice (ICH-GCP) E6 guidelines, the Declaration of Helsinki, the New Drugs and Clinical Trials Rules, 2019, and the Indian Council of Medical Research (ICMR) guidelines on clinical research. The protocol was approved by the Royal Pune Independent Ethics Committee on March 5, 2024. Written informed consent was obtained from all patients before participation, covering treatment details and follow-up procedures.

Inclusion and exclusion criteria

Adults (≥18 years) with T2DM who were administered dapagliflozin or an FDC containing dapagliflozin (dapagliflozin-metformin, dapagliflozin-sitagliptin, dapagliflozin-glimepiride-metformin, or dapagliflozin-sitagliptin-metformin) in any available dose were eligible for inclusion. Patients with type 1 diabetes mellitus, pregnant or breastfeeding women, and individuals with known hypersensitivity to dapagliflozin, metformin, sitagliptin, or glimepiride were excluded. Patients with severe hepatic or renal dysfunction at the time of dapagliflozin initiation were also excluded.

Data collection

Baseline demographic and clinical characteristics, diabetes history, dapagliflozin use (frequency, formulation, and dose), concomitant medical history, and laboratory parameters (glycated hemoglobin (HbA1c), fasting plasma glucose (FPG), and postprandial glucose (PPG)) were recorded. Patients were also asked to report the specific dapagliflozin and its combination used for their treatment.

Patients in the study were administered various formulations of dapagliflozin either as monotherapy or in FDC. These included dapagliflozin 5 and 10 mg as monotherapy; dapagliflozin combined with metformin extended-release (5 mg/500 mg, 5 mg/1000 mg, 10 mg/500 mg, and 10 mg/1000 mg) (Synokem Pharmaceuticals Limited, New Delhi, India); dapagliflozin with sitagliptin (5 mg/50 mg and 10 mg/100 mg), triple therapy with dapagliflozin (10 mg), sitagliptin (100 mg) with metformin extended-release (500 mg or 1000 mg) (Exemed Pharmaceuticals, Mumbai, India); and dapagliflozin with glimepiride (10 mg/1 mg or 10 mg/2 mg), both with metformin extended-release (500 or 1,000 mg) (Mascot Health Series Pvt. Ltd., Mumbai, India).

Endpoint

The primary endpoint was to assess the usage patterns of dapagliflozin and dapagliflozin FDCs (two-drug or three-drug combinations) at both the national and zonal levels across India. The secondary endpoints included evaluation of patient demographics, duration of T2DM, associated comorbidities, and glycemic profile among those receiving dapagliflozin therapies.

Data analysis

The statistical analysis was performed using SPSS software, version 23.0 (IBM Corp., Armonk, NY). Continuous variables were expressed as mean ± standard deviation, and categorical variables as percentages. The association between usage patterns of drugs for various HbA1c categories, comorbidities, and diabetes duration was tested using the chi-square test. Statistical significance was set as *P*-value < 0.01.

## Results

Baseline demographics

A total of 34,443 patients were enrolled in the study, of whom 19,501 (56.62%) were male. The mean ± SD age was 54.94 ± 11.35 years. The mean ± SD HbA1c was 8.53 ± 1.56%. The overall mean ± SD BMI, FPG, and PPG were 27.21 ± 4.93 kg/m², 176.66 ± 60.66 mg/dL, and 252.54 ± 83.44 mg/dL, respectively. Most patients (81.82%) had no history of alcohol use or smoking. The HbA1c levels ranged from 7% to 8% in 10,553 (30.64%) of patients, while 9,810 (28.48%) had HbA1c greater than 9%. About 23,011 (66.81%) of patients were obese (BMI > 24.9 kg/m²), and 17,044 (49.94%) had a diabetes duration of one to five years. Regarding kidney function, 551 patients (1.6%) had moderate impairment (stage 3a or 3b), and 541 patients (1.57%) had severe impairment or kidney failure (stage 4 or 5). The majority (26,779, 77.75%) had an unknown kidney function status. Common comorbidities included elevated blood pressure (120/70-139/89) (27,222, 79.03%), hypertension (11,599, 33.68%), dyslipidemia (11,431, 33.19%), CKD (10,562, 30.67%), and HF (8,615, 25.01%) (Table 2).

**Table 2:** Demographic characteristics. Data are presented as *n* (%), unless otherwise specified. ASCVD, atherosclerotic cardiovascular disease; BMI, body mass index; CKD, chronic kidney disease; FPG, fasting plasma glucose; HbA1c, glycated hemoglobin; HF, heart failure; PAD, peripheral artery disease; PPG, postprandial glucose

Parameters	No. of response, *n* (%) (*N* = 34,443)
Gender	
Male	19,502 (56.62)
Female	14,940 (43.38)
Other	1 (0.003)
Age (years), mean ± SD (years)	54.94 ± 11.35
Weight, mean ± SD (kg)	70.80 ± 11.38
Height (mean ± SD (cm)	161.88 ± 10.77
BMI (kg/m^2^), mean ± SD (kg/m^2^)	27.21 ± 4.93
HbA1c (%), mean ± SD	8.53 ± 1.56
FPG, mean ± SD (mg/dL)	176.66 ± 60.66
PPG, mean ± SD (mg/dL)	252.54 ± 83.44
Addiction status	
Alcohol user	1,566 (4.55)
Smoker	1,523 (4.42)
Both	3,172 (9.21)
None	28,182 (81.82)
HbA1c (%) level	
<7	5,272 (15.31)
7-8	10,553 (30.64)
8.1-9	8,806 (25.57)
>9	9,810 (28.48)
Hypertension (mmHg) stage	
Elevated (120/70-139/89)	27,222 (79.03)
Hypertension (>139/89)	6,275 (18.22)
Non-elevated (<120/70)	946 (2.75)
Associated conditions	
Hypertension (mmHg)	11,599 (33.68)
Dyslipidemia	11,431 (33.19)
CKD	10,562 (30.67)
HF	8,615 (25.01)
ASCVD	1,120 (3.25)
PAD	628 (1.82)
Stroke	582 (1.69)
BMI category	
Obese (>24.9)	23,011 (66.81)
Overweight (22.9-24.9)	5,664 (16.44)
Normal (18.5-22.9)	5,280 (15.33)
Underweight (<18.5)	488 (1.42)
CKD	
Unknown	26,779 (77.75)
Not CKD	6,539 (18.98)
Stage 1	12 (0.03)
Stage 2	21 (0.06)
Stage 3a	280 (0.81)
Stage 3b	271 (0.79)
Stage 4	193 (0.56)
Stage 5	348 (1.01)
Diabetes duration	
<1 year	1,795 (5.21)
1-5 years	17,044 (49.94)
6-10 years	11,992 (34.82)
>10 years	3,605 (10.47)

Geographic distribution of drug formulation use

**Figure 1:**
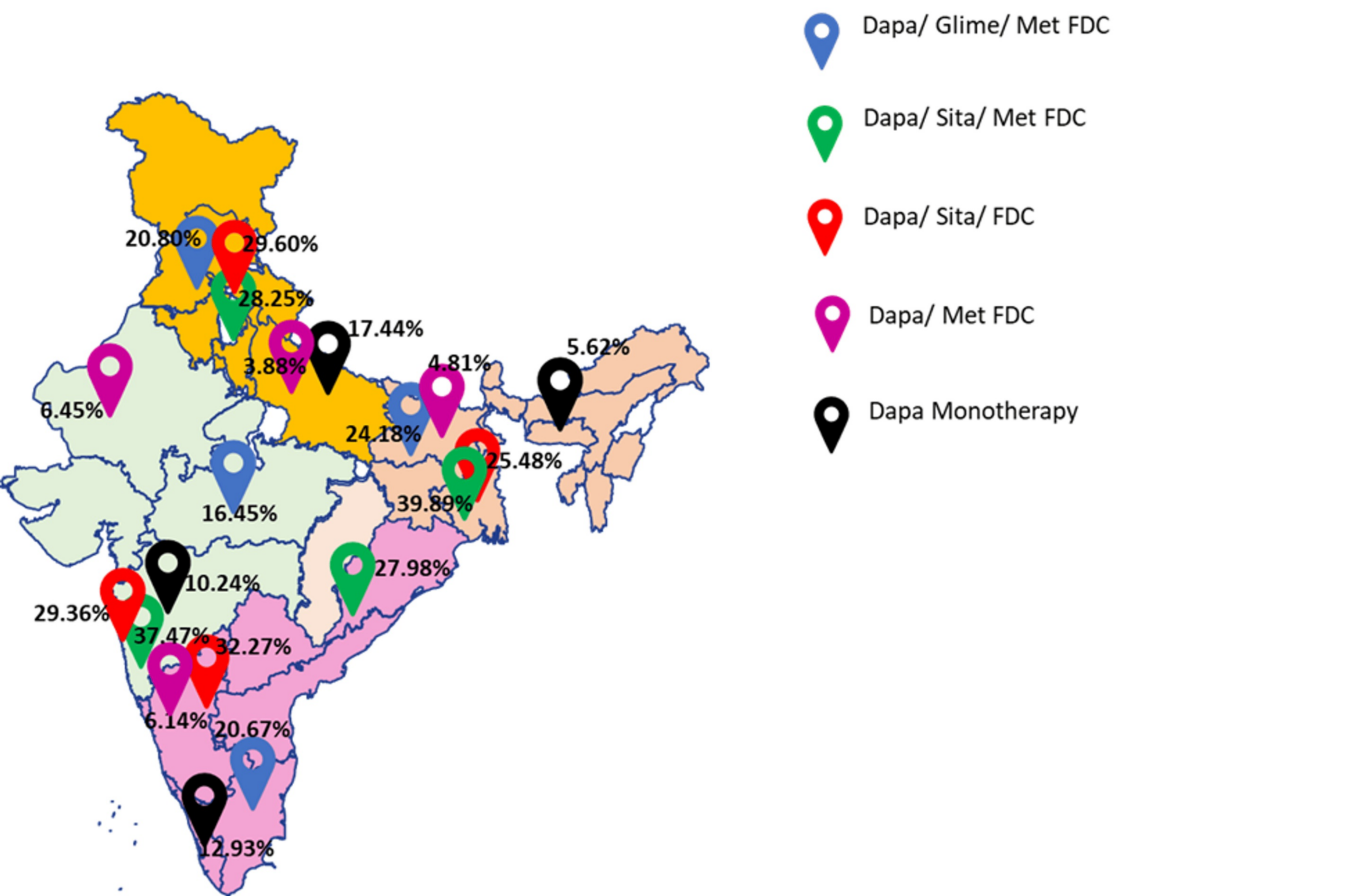
Zone-wise-distribution-based FDC usage (N = 34,443). This image highlights variations in prescribing patterns across the North, South, East, and West zones of India. The percentages shown for each zone correspond to the proportion of patients receiving different dapagliflozin-based regimens. Image credit: All authors. FDC, fixed-dose combination; Dapa, dapagliflozin; Glime, glimepiride; Met, metformin; Sita, sitagliptin

Usage patterns were consistent across all zones. The most widely used formulations were dapagliflozin + sitagliptin + metformin FDC (North, 28.25%; South, 27.98%; East, 39.89%; West, 37.47%) and dapagliflozin + sitagliptin FDC (North, 29.60%; South, 32.27%; East, 25.48%; West, 29.36%). The dapagliflozin + metformin FDC usage was North, 3.88%; South, 6.14%; East, 4.81%; West, 6.45%. The overall order of use was dapagliflozin + sitagliptin > dapagliflozin + sitagliptin + metformin > dapagliflozin + glimepiride + metformin > dapagliflozin + metformin > dapagliflozin (Figure 1, Table 3).

**Table 3:** Zone-wise-distribution-based FDC usage. Data are presented as *n* (%). FDC, fixed-dose combination

Zone	Dapagliflozin, glimepiride, and metformin triple FDC	Dapagliflozin, sitagliptin, and metformin triple FDC	Dapagliflozin, sitagliptin, dual FDC	Dapagliflozin and metformin dual FDC	Dapagliflozin monotherapy
North (*n* = 8,035)	1,672 (20.80)	2,270 (28.25)	2,379 (29.60)	312 (3.88)	1,402 (17.44)
South (*n* = 8,205)	1,696 (20.67)	2,296 (27.98)	2,648 (32.27)	504 (6.14)	1,061 (12.93)
East (*n* = 11,143)	2,695 (24.18)	4,445 (39.89)	2,840 (25.48)	536 (4.81)	627 (5.62)
West (*n* = 7,060)	1,162 (16.45)	2,646 (37.47)	2,073 (29.36)	456 (6.45)	723 (10.24)
Pan India (*n* = 34,443)	7,225 (20.98)	11,657 (33.84)	9,940 (28.86)	1,808 (5.24)	3,813 (11.07)

Comorbidity-specific drug formulation use

Overall, dapagliflozin + sitagliptin and dapagliflozin + sitagliptin + metformin FDCs were the most frequently used across comorbidities. Dapagliflozin monotherapy and dapagliflozin + glimepiride + metformin were used at moderate rates, while dapagliflozin + metformin consistently showed the lowest utilization. In patients with T2DM and comorbid conditions such as hypertension, dyslipidemia, atherosclerotic cardiovascular disease (ASCVD), and HF, dapagliflozin + sitagliptin was most commonly used, often in combination with telmisartan, rosuvastatin, and metoprolol (Table 4).

**Table 4:** Distribution of comorbidities based on drug formulation, diabetes duration, and HbA1c levels (N = 34,443). Data are presented as *n* (%). ASCVD, atherosclerotic cardiovascular disease; CKD, chronic kidney disease; FDC, fixed-dose combination; HbA1c, glycated hemoglobin; HF, heart failure

	Hypertension	Dyslipidemia	CKD	ASCVD	HF
Dapagliflozin-based FDC category					
Dapagliflozin and metformin dual FDC (*n* = 341)	129 (37.82)	76 (22.28)	11 (3.22)	112 (32.84)	13 (3.81)
Dapagliflozin and sitagliptin dual FDC (*n* = 2,069)	1,054 (50.94)	500 (24.16)	54 (2.60)	409 (19.76)	52 (2.51)
Dapagliflozin, sitagliptin, and metformin triple FDC (*n* = 1,712)	703 (41.06)	287 (16.76)	26 (1.51)	683 (39.89)	13 (0.75)
Dapagliflozin, glimepiride, and metformin triple FDC (*n* = 2,006)	741 (36.93)	388 (19.34)	47 (2.34)	782 (38.98)	48 (2.39)
Diabetes duration					
<1 year (*n* = 208)	44 (21.15)	45 (21.63)	3 (1.44)	115 (55.28)	1 (0.48)
1-5 years (*n* = 3,664)	1,422 (38.81)	613 (16.73)	96 (2.62)	1,433 (39.11)	100 (2.72)
6-10 years (*n* = 2,197)	1,177 (53.57)	512 (23.30)	35 (1.59)	437 (19.89)	36 (1.63)
>10 years (*n* = 1,032)	412 (39.92)	255 (24.70)	12 (1.16)	345 (33.43)	8 (0.77)
HbA1c levels					
HbA1c < 8.5% (*n* = 3,801)	1,616 (42.51)	789 (20.75)	81 (2.13)	1,228 (32.30)	87 (2.28)
HbA1c > 8.5% (*n* = 3,301)	1,440 (43.62)	636 (19.26)	65 (1.96)	1,102 (33.38)	58 (1.75)

**Figure 2:**
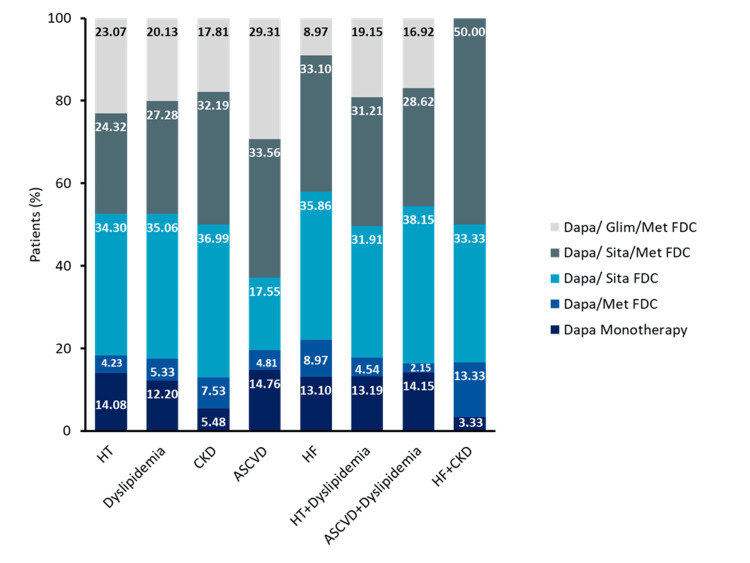
Dapagliflozin drug formulation in patients with T2DM based on different comorbidities (N = 34,443). ASCVD, atherosclerotic cardiovascular disease; CKD, chronic kidney disease; FDC, fixed-dose combination; HF, heart failure; HT, hypertension; Dapa, dapagliflozin; Glime, glimepiride; Met, metformin; Sita, sitagliptin

In patients with T2DM and multiple comorbidities, the use of dapagliflozin + sitagliptin + metformin FDC was significantly higher among those with both HF and CKD (50%) compared with other conditions (*P *< 0.001) (Figure 2, Table 5).

**Table 5:** Chi-square test result; dapagliflozin drug formulation usage pattern. *Test of independence was used to derive the chi-square statistics. HbA1c, glycated hemoglobin

Usage pattern	Degree of freedom	Chi-square statistics^*^	*P*-value
Different comorbidities	35	325.35	<0.001
HbA1c level	4	295.97	<0.001
Diabetes duration	12	315.13	<0.001

HbA1c-wise comorbidities and specific drug formulation use

**Figure 3:**
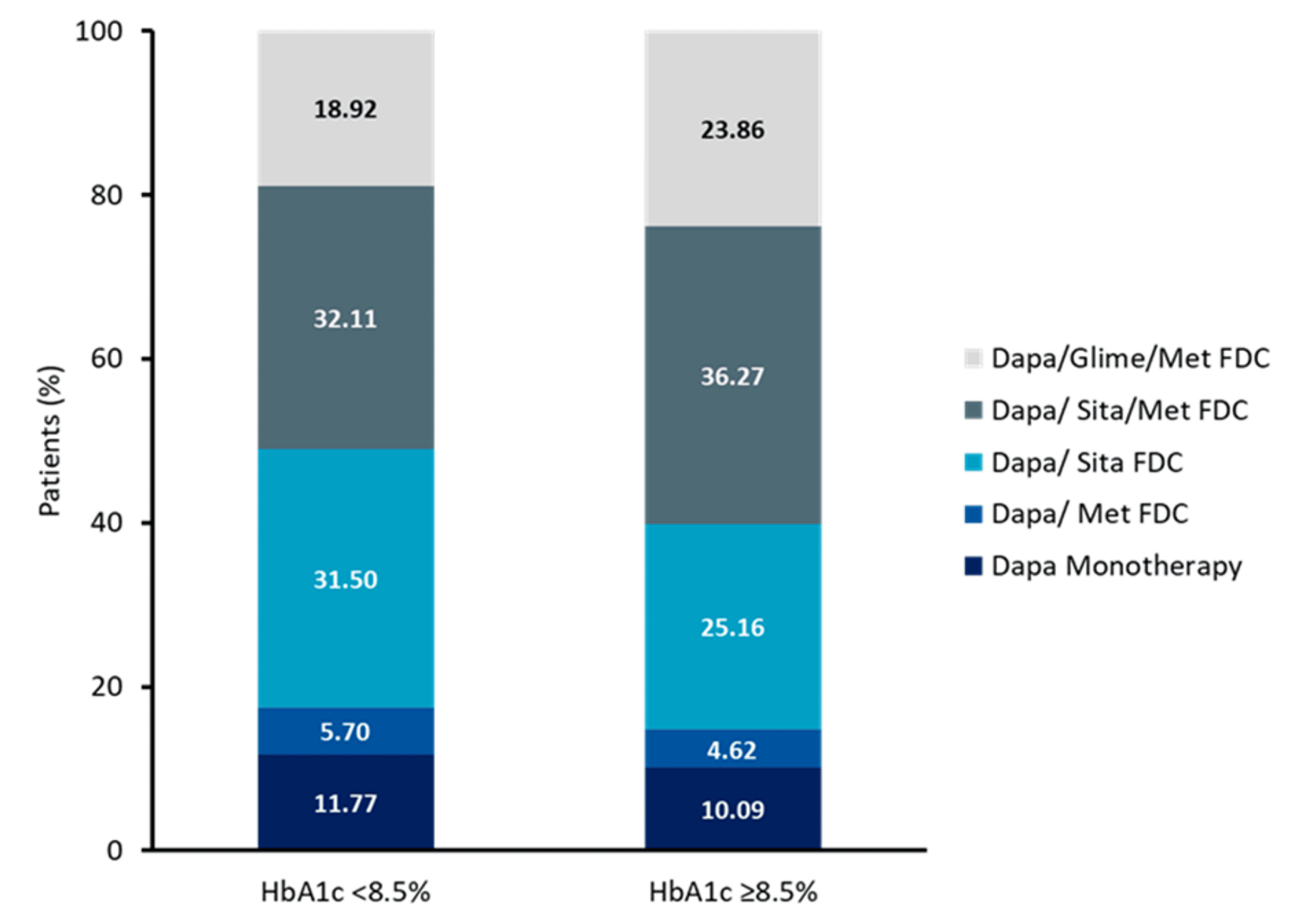
Dapagliflozin drug formulation based on HbA1c level (N = 34,443). FDC, fixed-dose combination; HbA1c, glycated hemoglobin; Dapa, dapagliflozin; Glime, glimepiride; Met, metformin; Sita, sitagliptin

The association of hypertension and ASCVD was more frequent in both HbA1c groups (<8.5% and ≥8.5%) compared with other comorbid conditions (Table 4). Among antidiabetic therapies, dapagliflozin + sitagliptin (31.50% vs. 25.16%; *P* < 0.001) was the most frequently used in HbA1c <8.5% compared to those with HbA1c ≥8.5% (Figure 3, Table 5).

Diabetes duration-wise comorbidities and specific drug formulation use

Among patients with diabetes for a duration of 6 to >10 years, hypertension was frequently associated with comorbidity, whereas ASCVD occurred from a duration of <1 to 5 years (Table 4).

**Figure 4:**
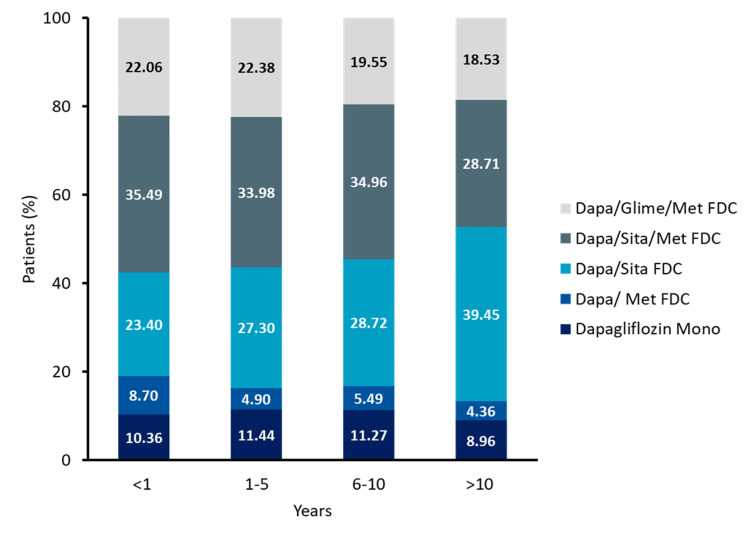
Dapagliflozin drug formulation usage pattern based on duration of diabetes (N = 34,443). FDC, fixed-dose combination; Dapa, dapagliflozin; Glime, glimepiride; Met, metformin; Sita, sitagliptin

Use of dapagliflozin + sitagliptin increased progressively with longer diabetes duration, from 23.4% in patients with <1 year of diabetes to 39.45% in those with >10 years (*P *< 0.001). Dapagliflozin + sitagliptin + metformin was consistently used across all groups, ranging from 35.49% in patients with <1 year of diabetes to 34.96% in those with 10 years (Figure 4, Table 5).

Co-prescribed medications

Patterns of co-prescription varied according to diabetes duration and HbA1c category. Dapagliflozin + sitagliptin was most frequently prescribed in patients with hypertension (50.94%), often with telmisartan, rosuvastatin, and metoprolol. The same dual FDC was also common in patients with dyslipidemia (24.16%) and HF (92.51%). In patients with ASCVD (39.89%), dapagliflozin + glimepiride + metformin FDC was most often prescribed.

## Discussion

This real-world study evaluated the clinical characteristics and treatment patterns of patients with T2DM receiving dapagliflozin monotherapy or FDCs across India. The frequent use of dual and triple combinations of dapagliflozin and sitagliptin provides complementary mechanisms of action and suggests a strong rationale for their widespread adoption in Indian clinical practice.

The mean age of patients in this study was 54.94 years. The mean baseline HbA1c, FPG, and PPG were 8.53, 176.66, and 252 mg/dL, respectively. In comparison, the ADMIRE real-world study reported a higher mean age (59.7) and HbA1c (9.34), with lower baseline FPG (159.1 mg/dL) and PPG (239.3 mg/dL) [11]. These findings suggest that patients in this study cohort were younger and had moderately elevated glycemic parameters compared with ADMIRE, indicating a potentially earlier stage of T2DM presentation in Indian outpatient practice.

A considerable proportion of patients had suboptimal glycemic control (HbA1c >7%), with obesity and hypertension being the most frequent comorbidities. In another study, HbA1c levels of 7%-8% were reported in 25.1% and >9% in 37.7%, while hypertension and obesity were present in 60.5% and 82.4%, respectively [12]. The consistently high prevalence of obesity and hypertension across studies underscores the importance of multidrug regimens that address not only hyperglycemia but also cardiometabolic risk factors. In our cohort, hypertension (33.68%) and dyslipidemia (33.19%) were common, in line with findings from other real-world settings [13]. Such coexistence of cardiometabolic comorbidities highlights the need for comprehensive management strategies when selecting antidiabetic therapies.

Across regions, dapagliflozin + sitagliptin and dapagliflozin + sitagliptin + metformin were the most frequently used formulations. The preference likely reflects their proven efficacy in controlling both fasting and postprandial glucose, physician familiarity, and supportive evidence showing superior outcomes with dual and triple therapy compared with stepwise monotherapy. The increasing use of dapagliflozin + sitagliptin in patients with longer diabetes duration further highlights the progressive nature of T2DM and the need for timely intensification [14]. In the Indian context, SGLT2 inhibitors + DPP-4 inhibitors combinations are considered a particularly effective strategy, offering durable glycemic control through complementary mechanisms, while addressing unique challenges such as earlier disease onset, prominent postprandial hyperglycemia, and high cardiometabolic risk. These regimens also provide added benefits of weight and blood pressure reduction with a low risk of hypoglycemia, making them well-suited for routine clinical practice [15].

In patients with comorbid conditions, such as hypertension, dyslipidemia, CKD, HF, or ASCVD, dapagliflozin + sitagliptin was the most commonly used. A similar trend was reported in another study, where patients with a higher prevalence of cardiovascular disease and HF received sitagliptin + dapagliflozin [16]. This suggests that physicians may be prioritizing sitagliptin-based combinations for their established efficacy, tolerability, and suitability in high-risk populations.

Because a large proportion of patients had HbA1c greater than 7%-8%, sitagliptin-containing combinations were more preferred to achieve glycemic targets. This aligns with prior studies suggesting that add-on therapy with dapagliflozin in patients already receiving sitagliptin (with or without metformin) improves glycemic control in those with suboptimal HbA1c [17].

In this study, the use of dapagliflozin + sitagliptin increased progressively with diabetes duration, from 23.4% in patients with <1 year of diabetes to 39.45% in those with >10 years. Dapagliflozin + sitagliptin + metformin was consistently used across all groups (35.49% in <1 year vs. 34.96% in >10 years). Comparable trends have been observed in other real-world studies, where triple therapy was more frequently prescribed (46.71%) among patients with longer disease duration [16]. This reflects clinicians’ tendency to escalate therapy in line with the progressive nature of T2DM and declining β-cell function.

Strengths and limitations

This study was conducted across multiple healthcare centers in India, capturing real-world prescribing practices beyond the controlled settings of randomized clinical trials. The large sample size provides robust evidence supporting the use of dapagliflozin FDCs in Indian outpatient care. However, reliance on case record forms without accounting for lifestyle factors limits the ability to fully assess determinants of glycemic control. Furthermore, follow-up data on adherence and long-term outcomes, including safety, were unavailable.

## Conclusions

This study highlights real-world treatment patterns of dapagliflozin use in India, demonstrating that combination regimens, particularly with sitagliptin, are widely adopted across diverse patient groups. These findings emphasize the role of complementary therapies in addressing the complex clinical profiles of patients with T2DM and provide a framework for understanding treatment practices in routine care.
